# Vector composition, abundance, biting patterns and malaria transmission intensity in Madang, Papua New Guinea: assessment after 7 years of an LLIN-based malaria control programme

**DOI:** 10.1186/s12936-021-04030-4

**Published:** 2022-01-05

**Authors:** John B. Keven, Michelle Katusele, Rebecca Vinit, Daniela Rodríguez-Rodríguez, Manuel W. Hetzel, Leanne J. Robinson, Moses Laman, Stephan Karl, Edward D. Walker

**Affiliations:** 1 Department of Entomology, Michigan State University, East Lansing, MI USA; 2 Department of Microbiology and Molecular Genetics, Michigan State University, East Lansing, MI USA; 3 Papua New Guinea Institute of Medical Research, Vector-Borne Diseases Unit, Madang, Papua New Guinea; 4 Swiss Tropical and Public Health Institute, Basel, Switzerland; 5 University of Basel, Basel, Switzerland; 6 Burnet Institute, Melbourne, VIC Australia; 7 Walter and Eliza Hall Institute of Medical Research, Parkville, VIC Australia; 8 Department of Medical Biology, University of Melbourne, Melbourne, VIC Australia; 9 Australian Institute of Tropical Health and Medicine, James Cook University, Cairns, QLD Australia

**Keywords:** Abundance, *Anopheles*, Composition, Malaria, Mosquitoes, Transmission, Vectors

## Abstract

**Background:**

A malaria control programme based on distribution of long-lasting insecticidal bed nets (LLINs) and artemisinin combination therapy began in Papua New Guinea in 2009. After implementation of the programme, substantial reductions in vector abundance and malaria transmission intensity occurred. The research reported here investigated whether these reductions remained after seven years of sustained effort.

**Methods:**

All-night (18:00 to 06:00) mosquito collections were conducted using human landing catches and barrier screen methods in four villages of Madang Province between September 2016 and March 2017. *Anopheles* species identification and sporozoite infection with *Plasmodium vivax* and *Plasmodium falciparum* were determined with molecular methods. Vector composition was expressed as the relative proportion of different species in villages, and vector abundance was quantified as the number of mosquitoes per barrier screen-night and per person-night. Transmission intensity was quantified as the number of sporozoite-infective vector bites per person-night.

**Results:**

Five *Anopheles* species were present, but vector composition varied greatly among villages. *Anopheles koliensis*, a strongly anthropophilic species was the most prevalent in Bulal, Matukar and Wasab villages, constituting 63.7–73.8% of all *Anopheles*, but in Megiar *Anopheles farauti* was the most prevalent species (97.6%). Vector abundance varied among villages (ranging from 2.8 to 72.3 *Anopheles* per screen-night and 2.2–31.1 *Anopheles* per person-night), and spatially within villages. Malaria transmission intensity varied among the villages, with values ranging from 0.03 to 0.5 infective *Anopheles* bites per person-night. Most (54.1–75.1%) of the *Anopheles* bites occurred outdoors, with a substantial proportion (25.5–50.8%) occurring before 22:00.

**Conclusion:**

The estimates of vector abundance and transmission intensity in the current study were comparable to or higher than estimates in the same villages in 2010–2012, indicating impeded programme effectiveness. Outdoor and early biting behaviours of vectors are some of the likely explanatory factors. Heterogeneity in vector composition, abundance and distribution among and within villages challenge malaria control programmes and must be considered when planning them.

**Supplementary Information:**

The online version contains supplementary material available at 10.1186/s12936-021-04030-4.

## Background

In Papua New Guinea (PNG), all four solely human malaria parasite species are found, but *Plasmodium falciparum* and *P. vivax* are the most prevalent and clinically important [1–3]. The *Plasmodium* parasites are transmitted mainly by members of the *Anopheles punctulatus* group of species [4]. Of the 13 closely related species comprising this group, *Anopheles farauti *sensu stricto (*s.s*.), *An. koliensis* and *An. punctulatus s.s*. are the primary vectors [5–12]. Two species outside this group, *An. bancroftii* and *An. longirostris*, are often found in sympatry with members of the punctulatus group. However, they are secondary vectors, primarily because they are often present in low numbers and *An. bancroftii* is zoophilic [4]. These vector species are often found together in mosquito samples from a locality (e.g., village), but their relative composition in a sample can vary greatly. Variation in vector composition is associated with geographic distribution of the *Anopheles* species. *An. farauti s.s*. is found most frequently in the outer islands and along the coastal plains of mainland PNG [13, 14]. Its abundance relative to other *Anopheles* species diminishes rapidly beyond 1 km from the shoreline [13, 14]. *An. punctulatus s.s.* and *An. koliensis* are often present in samples from the coast, however, they are most abundant in inland areas beyond 1 km from the shoreline [13, 14]. *An. punctulatus s.s.* tends to be more abundant than *An. koliensis* in hilly areas whereas in lowland areas *An. koliensis* tends to be more abundant then *An. punctulatus s.s.* [15].

A considerable reduction in the worldwide burden of malaria has been achieved over the past two decades as a consequence of vector control methods, particularly the use of long-lasting insecticidal bed nets (LLINs) and indoor residual sprays, as well as increased availability of anti-malarial drugs and rapid diagnostic tests [16–18]. Despite this global success, malaria continues to be an important and intractable public health problem in many developing tropical countries, including PNG [16–18]. In the period preceding 2009, the year a national malaria control programme was implemented in PNG, prevalence of malaria infection based on microscopy diagnosis of blood samples in human populations in the coastal and inland lowland areas (below 600 m altitude), particularly Madang and East Sepik provinces, varied from < 10 to > 70% amongst sites, but tended to equilibrate between 35 and 45% [8, 19, 20]. Annual entomological inoculation rate (EIR), a measure of malaria transmission intensity, ranged from 68 to 526 sporozoite-infective *Anopheles* bites per person-year [9]. It was estimated that 4–17% of deaths in children under 10 years old in PNG were caused by malaria [21, 22]. Beginning in 2009, a malaria control programme was implemented nationwide by the PNG National Department of Health with the financial backing of international donors including the Global Fund. The control programme involved free distribution of pyrethroid-impregnated LLINs as the primary control method, supplemented with increased supply of artemisinin combination therapy (ACT) and rapid diagnostic test kits at local health centres throughout the country [23–26]. Deltamethrin-treated Permanet® 2.0 (Vestergaard–Frandsen) was the brand of LLINs exclusively distributed in PNG [27]. As malaria transmission occurs when humans are exposed to the bites of sporozoite-carrying female *Anopheles*, LLINs reduce exposure to infective bites by serving as a physical barrier between humans and mosquitoes, and by reducing vector abundance and lifespan through lethal, physical contact [28–31]. At health centres, rapid diagnostic tests help to ascertain the infection status of a patient, and the artemisinin combination therapy is administered to clear the parasites from a patient’s body. Concurrent with roll-out of LLINs was a considerable decline of infection prevalence in humans and transmission intensity of vectors nationally [11, 32–35]. However, the downward trend of malaria did not continue; a 2016–2017 national survey found a nine-fold increase in infection prevalence (any malaria species) compared to the prevalence estimates in a 2013–2014 survey [36]. In coastal villages of Madang Province, the infection prevalence of *P. falciparum* in 2017 (19.1–28.3%) increased by *ca.* two-fold compared to 2014 estimates (11.4–12.3%). *P. vivax* prevalence in Madang villages remained steady between the two years but was high (18.3–23.4%) [37, 38].

The persistence and resurgence of malaria in PNG could be caused by several factors. Although decline in the use of LLINs is one, nationwide surveys of LLIN usage revealed steady or increasing use of LLINs between 2008 and 2017 [33, 36]. In the coastal villages of Madang, > 80% of village residents interviewed in 2016 or 2017 reportedly use LLINs regularly [39]. Shortage in the supply of anti-malarial drugs is unlikely to be the cause of malaria resurgence in PNG considering that > 80% of infections in humans are asymptomatic [35, 40] and anti-malarials are administered only to patients with clinical symptoms who present at local health centres. However, nearly 50% of anti-malarials (particularly primaquine) in the supply chain were sub-standard and thus may not achieve satisfactory clinical outcomes nor reduce transmission potential [41]. Anti-malarial resistance to non-ACT is prevalent in PNG long before the malaria control programme began. However, malaria parasites (all species) in PNG are still susceptible to ACT (first-line treatment in PNG), although presence of an ACT-resistant mutation has been recently detected by genetic screening in some *P. falciparum* isolates in PNG [42, 43]. Factors that reduce vectors’ risk of exposure to or mortality from the LLINs are potential causes of malaria resurgence in PNG. Physiological resistance to the pyrethroids in the LLINs is one such factor but it has not been detected in PNG vectors so far, including in *Anopheles* populations near or in the current study region [44–46]. However, there is evidence that the LLINs distributed between 2013 and 2019 in PNG had low bioefficacy against natural populations of susceptible vectors as well as colonized mosquitoes [27]. Behavioural factors, such as tendency of vectors to bite humans outdoors where bed nets do not offer protection, and early in the evening when most people are awake and unprotected by the bed nets could also obviate effectiveness of LLINs, allowing vectors to bite humans and at the same time evade exposure to them [47–49].

Given the above background, the objective of this study was to investigate *Anopheles* vector composition, abundance, rate of biting on humans, spatial and temporal biting patterns, and transmission intensity of malaria in villages in a coastal area of Madang Province, PNG. These villages had high rates of LLIN use (> 80% of residents use bed nets) [39]. Prevalence of infection in humans was also surveyed in parallel to the current study and the results are presented elsewhere [37, 38]. Given the nine-fold increase in malaria infection prevalence in a national survey in 2016–2017 [36], transmission intensity was expected to be higher in the current study compared to studies conducted immediately after the LLIN programme.

## Methods

### Study sites

**Fig. 1:**
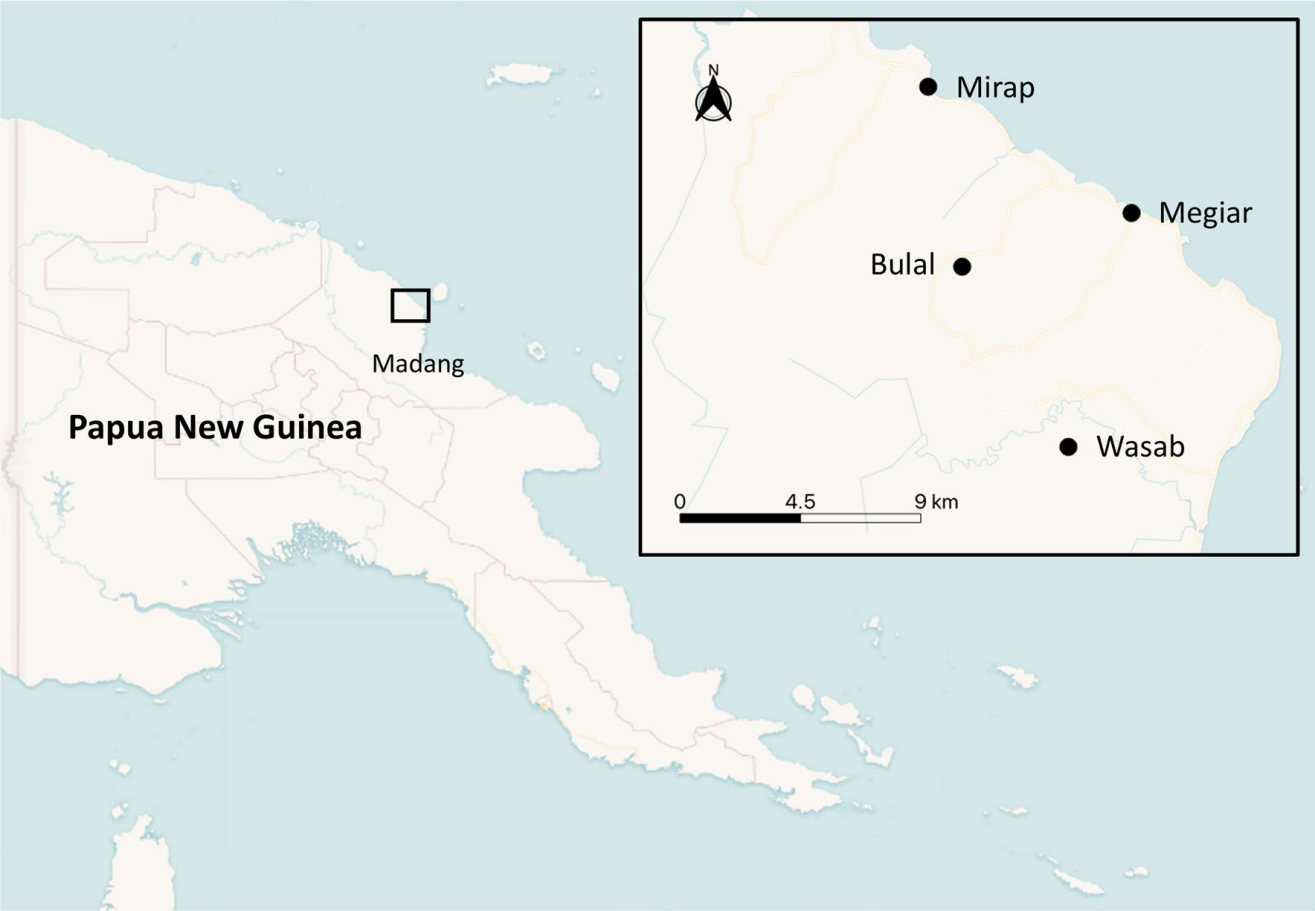
Map showing the location of the four study villages in Madang province, Papua New Guinea

This study was conducted between September 2016 and March 2017 in Bulal, Megiar, Mirap and Wasab villages in the north coast of Madang Province, PNG (Fig. 1), an historically endemic region [3, 19, 50]. Megiar and Mirap are situated on the coastal plain about 2–4 m above sea level, whereas Bulal and Wasab are located several km inland from the coast, on elevated hilltops about 150 m above sea level. The landscape and vegetation of the coastal and inland environments where the villages are located are described elsewhere [51, 52]. Like most other coastal areas of PNG, the average monthly rainfall in the study region ranged from 250 to 350 mm. The wet season occurs from October-May and dry season from June–September (https://climateknowledgeportal.worldbank.org/country/papua-new-guinea).

### Mosquito sampling

Host-seeking female mosquitoes were collected using two methods: human landing catch (HLC) and barrier screen sampling (BSS). The HLC method involved human volunteers capturing mosquitoes that landed on exposed parts of their legs using a mouth aspirator, aided by a hand-held flash light to see the resting mosquitoes on their legs [53, 54]. In each village, 24 (Megiar, Mirap and Wasab) or 20 (Bulal) houses were selected to participate in the HLC mosquito collections. The houses were randomly divided into two groups with equal number of houses in each group. For houses in one group, mosquitoes were collected next to sleeping spaces inside the houses, hereafter referred to as indoor collections. For houses in the second group, mosquitoes were collected outside but near the houses, hereafter referred to as outdoor collections. For each house, two members (18–70 years old) of a family were consented and trained to collect mosquitoes inside or outside their own house. All houses in which mosquitoes were sampled were temporary ones built using bush materials either on the ground or raised above ground on short posts (0.5–1.0 m high). These are the most common types of houses in the study villages; only a few families owned permanent or semi-permanent houses. The BSS method involved search and collection of mosquitoes that rested on the surface of the barrier screens. Description of the structure and set-up of the barrier screen equipment is presented in detail elsewhere [52, 55, 56]. Briefly, a barrier screen consisted of a 20-m long polyethylene shade cloth (70% shading) fastened to wooden poles and erected vertically to a height of 2 m [52]. The barrier screens were positioned at locations near the village perimeter between the village and the surrounding vegetations. Mosquitoes that rested on the surface of the barrier screens as they commute into and out of the village were captured by mosquito collectors [52]. In the current study, mosquitoes were sampled with eight barrier screens each night in each village. For each barrier screen, two local volunteers were consented and trained to search and collect resting mosquitoes. The procedure for searching and collecting mosquitoes that rested on the barrier screens is described in length elsewhere [51, 52]. While the mosquito collectors for the barrier screen method were male volunteers, the HLC collectors consisted of equal proportion of male and female volunteers.

For both sampling methods, all-night (18:00 to 06:00 h) mosquito collections were conducted for four consecutive nights in Bulal (March 16–19, 2017), six nights in Megiar (February 4–9, 2017) and Mirap (January 11–16, 2017) and 12 nights in Wasab (September 5–10 and November 4–9, 2016). Mosquitoes were collected by both sampling methods simultaneously in each village. However, not all HLC houses were sampled simultaneously every night. In three of the villages (Megiar, Mirap, Bulal), mosquitoes were collected for two nights in each house. In Wasab, mosquitoes were collected for four nights in each house. For the BSS collections in all the villages, mosquitoes were sampled at each barrier screen every night. At each barrier screen or house, one of the two volunteers collected mosquitoes for the first 6 h (18:00 to 00:00) before being replaced by the second volunteer who continued for the next 6 h (00.00 to 06:00). Captured mosquitoes were placed into screened paper cups pre-labelled with the hour of the night and the house or barrier screen number. Upon the next morning and with the aid of a light microscope, mosquitoes were separated into their respective genera. Each female *Anopheles* mosquito was morphologically identified to species [57, 58], placed in a 2-ml microcentrifuge tube, and assigned a unique identification number. Metadata (morphospecies, village, house or barrier screen number, date, time of capture) associated with each mosquito identifier were recorded. The mosquitoes were kept on silica gel desiccant in the field for up to 7 days and then transported to the laboratory where they were stored at − 20 °C.

### Molecular identification of *Anopheles* species

Using sterile technique, the abdomen of each *Anopheles* mosquito was separated from the rest of the body and DNA was extracted from the abdomen-detached body part (i.e., head and thorax) using DNeasy Blood and Tissue Kit (Product number: 69582; Qiagen, Valencia, CA, USA). Mosquitoes that were morphologically identified as members of the punctulatus group were analysed using a standard polymerase chain reaction (PCR) method [59]. The PCR method involved amplification of the internal transcribed spacer region 2 of the ribosomal ribonucleic acid (rRNA) gene followed by fragmentation of the PCR amplicons with the restriction endonuclease MspI. The fragmented amplicons were then visualized on 2% ethidium bromide-stained agarose gel to determine the species of the *Anopheles* based on band pattern of the DNA fragments.

### Molecular detection of sporozoites in mosquitoes

A multiplex quantitative PCR with two fluorescent-labelled TaqMan probes targeting the 18S rRNA gene of *P. falciparum* (forward primer: ATT GCT TTT GAG AGG TTT TGT TAC TTT; reverse primer: GCT GTA GTA TTC AAA CAC AAT GAA CTC AA; probe: FAM-CAT AAC AGA CGG GTA GTC AT-MGB) and *P. vivax* (forward primer: GCA ACG CTT CTA GCT TAA TCC AC; reverse primer: CAA GCC GAA GCA AAG AAA GTC C; probe: VIC-ACT TTG TGC GCAT TTT GCT A-MGB) was optimized using the same method described for blood-meal quantitative PCR [60]. The primers and probes were designed and tested to be specific to the target malaria species and gene locus by Kamau et al. [61]. Ten-fold dilution series of positive DNA controls of both malaria species were used for optimization of the assays. The PCR reaction mixtures (10 μl final volume) consisted of 1 × TaqMan PCR master mix (Product number: 4461882; Thermo Fisher Scientific, Waltham, MA, USA), 0.6 μM of each primer, 0.4 μM of each probe and 2 μl of DNA samples (10^–5^–10 ng/μl). PCR reactions were performed on a QuantStudio 7 Flex instrument (Applied Biosystems, Foster City, CA, USA) with fast cycling conditions (1 cycle of 95 °C for 20 s followed by 40 cycles of 95 °C for 1 s and 60 °C for 20 s). PCR sensitivity was one target gene copy/μl sample, and its amplification efficiency was > 90%.

Mosquito DNA was analysed using the quantitative PCR method to test for *Plasmodium* infection in the mosquitoes. Only *Anopheles* from HLC were tested for infections. Samples with amplification threshold cycles ≥ 38 were considered inconclusive and therefore negative. As the mosquito DNA was isolated from part of the body anterior to the abdomen, it was considered devoid of oocysts and other human stages of the malaria parasites that might have been present in the midgut. Thus, the PCR-positive mosquitoes were assumed to carry the infective sporozoite stage which inhabits the salivary glands in the head and thorax [62].

### Data analysis

The composition of vectors in a village was expressed as the proportion of each vector species in a sample of *Anopheles* mosquitoes from that village. Variation in vector composition among villages or sampling location was tested using Chi-square analysis of contingency tables with vector species along the rows and villages or environments along the columns.

Collections conducted at one house over the course of one night were equivalent to one person-night. Based on the number of houses and nights of sampling at each house in the villages, a total of 48 person-night sampling replicates were generated in Megiar and Mirap, 40 person-night replicates in Bulal, and 96 person-night replicates in Wasab. Similarly, based on the number of barrier screens and nights of sampling at each screen in the villages, a total of 48 screen-night sampling replicates were generated in Megiar and Mirap, 32 screen-night replicates in Bulal, and 96 screen-night replicates in Wasab. Biting rates (number of mosquitoes per person-night) and resting rates (number of mosquitoes per screen-night) were calculated and used as measures of vector abundance in the villages. The term ‘biting rate’ is used here as it was assumed that the number of mosquitoes landing on a collector equates to the number of mosquito bites taken on the collector had the mosquitoes been provided the opportunity to bite before capture. The non-parametric Kruskal–Wallis rank sum analysis was used to test for variation in resting rates and biting rates among villages.

In each village, variation in the proportion of total HLC mosquitoes among three periods of the night (18:00–22:00, 22:00–02:00 and 02:00–06:00, corresponding to evening, late night and early morning periods) was tested using goodness-of-fit Chi-square analysis, with expected probability of 0.33 for all three test categories. The proportion of mosquitoes in indoor and outdoor collections were also calculated and goodness-of-fit Chi-square analysis was used to test for variation between the two categories, with expected probability of 0.5 for both test categories. The use of Chi-square analysis to evaluate variation in mosquito proportion between indoor and outdoor collections and among the three periods of the night was appropriate because of balanced mosquito sampling effort among the test categories.

Within a village, each house in which mosquitoes were collected represented a spatial unit of sampling (replicate). As all the sampled houses in a village had equal number of nights during which mosquitoes were collected (balanced sampling effort), the frequency distribution of mosquitoes in houses was analysed to characterize patterns of spatial distribution of vectors. The analysis was performed for indoor and outdoor collections separately using the index of dispersion, a quantity of the ratio of variance to the mean of the data. After calculating it, the estimated value was tested for departure from unity using Chi-square analysis: $\chi^{2} = \left( {\mathop \sum \nolimits_{i = 1}^{n} \left( {x_{i} - \overline{x}} \right)^{2} } \right)/\overline{x}$ with degrees of freedom of *n*–1 [63]. In the equation, *x*_*i*_ is the number of mosquitoes in *ith* house, $\overline{x}$ is the mean number of mosquitoes (averaging across houses), and *n* is the number of houses. The frequency distribution of mosquitoes fit a random distribution if the index of dispersion did not significantly deviate from 1, a uniform distribution if the index was significantly < 1, or a clustered (heterogeneous) distribution if it was significantly > 1 [63].

Sporozoite rate was quantified as the proportion of PCR-tested mosquitoes that were positive for malaria parasites. Malaria transmission intensity was expressed in terms of the nightly EIR (number of infective vector bites per person-night) and was quantified as follows. The total number of mosquitoes collected in each house was divided by the number of nights of collection in the house. As the number of houses in a village is the same as the number of HLC collectors in the village, the calculations described above yielded the nightly biting rate for each collector (number of vector bites that a collector receives per night). For each HLC collector, the number of infective vector bites encountered in a night was estimated by taking the product of two quantities: the nightly biting rate and the sporozoite rate. Nightly EIR was calculated by taking the mean infective vector bites with collector as unit of replication.

All the statistical analyses described above were performed in R software version 3.4.2 (https://www.r-project.org/). The Chi-square and Kruskal–Wallis tests were performed using the functions *chisq.test*, and *kruskal.test*, respectively, of the R package *stats*. Significance level of all statistical tests was based on type I error rate of 0.05.

## Results

### Vector composition

**Fig. 2:**
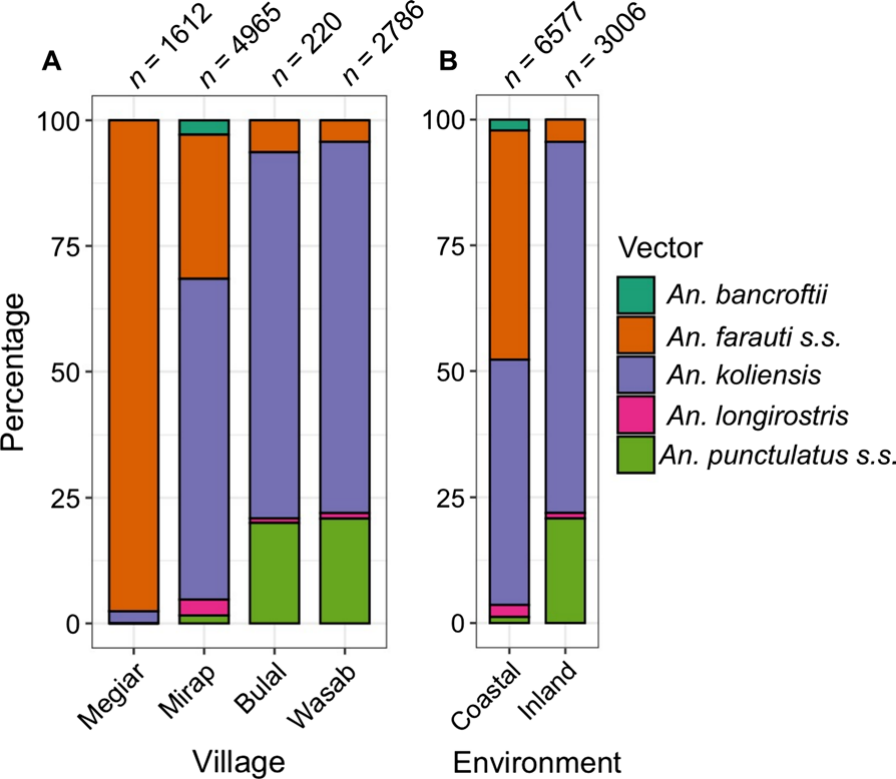
Stacked bar plots showing the proportion of vector species in samples of *Anopheles* mosquitoes from five different villages (**A**) and two ecogeographical environments (**B**). Mosquito sample size (*n*) for each village or environment is shown above the bar corresponding to the village or environment

A total of 9583 *Anopheles* mosquitoes were collected by both sampling methods combined. Of these, 142 (1.5%) were *An. bancroftii*; 3130 (32.7%) were *An. farauti s.s*.; 5417 (56.5%) were *An. koliensis*; 190 (2.0%) were *An. longirostris*; and, 704 (7.4%) were *An. punctulatus s.s*.. The number and percentages of each *Anopheles* species in mosquito samples from each village are presented in Additional file 1: Table S1 and Fig. 2A. The composition of *Anopheles* species in mosquito samples (excluding non-anophelines) within villages was not homogeneous (Fig. 2A). In Megiar, *An. farauti s.s.* and *An. koliensis* were the only species present in the sample, but the former species constituted the most (97.6%). In Mirap, all five species were present in the sample, however, most of the mosquitoes were *An. koliensis* (63.7%) followed by *An. farauti s.s*. (28.7%); the other three species together constituted only 7.6% of the sample. In Bulal and Wasab, all species except *An. bancroftii* were present. In both villages most of the mosquitoes in the samples were *An. koliensis* (Bulal, 72.7%; Wasab, 73.8%) and *An. punctulatus s.s*. (Bulal, 20%; Wasab, 20.8%); *An. farauti s.s.* or *An. longirostris* each constituted ≤ 6.36% of the sample. The relative proportion of *Anopheles* species in mosquito samples varied significantly among the villages (χ^2^ = 5167.9, df = 12, P < 0.001).

Mosquito data from the villages located in coastal plains (Megiar and Mirap) were combined and those from the villages in inland environment (Bulal and Wasab) were combined. The number and percentage of each vector species in the samples from the two environments are shown in Additional file 1: Table S2 and Fig. 2B. The relative proportion of the vectors varied significantly between the two environments (χ^2^ = 2457.4, df = 4, P < 0.001). In the coastal plain sample, *An. farauti s.s.* (45.6%) and *An. koliensis* (48.7%) almost equally constituted the majority; the other three vector species together constituted only a small fraction (5.8%) (Fig. 2B). Interestingly, most (98.8%, *n* = 3202) of the *An. koliensis* mosquitoes in the coastal sample were from Mirap, even though sampling effort was nearly the same in both coastal villages. In contrast, a large fraction of the mosquito sample from inland environment were *An. koliensis* (73.7%); *An. punctulatus s.s.* constituted 20.8% and the other three species together constituted only a small fraction (5.5%) (Fig. 2B). Generally, of the three primary vector species, *An. farauti s.s.* was associated with the coastal plains, *An. punctulatus s.s.* with the inland environment and *An. koliensis* with both environments.

### Resting and biting rates

**Fig. 3:**
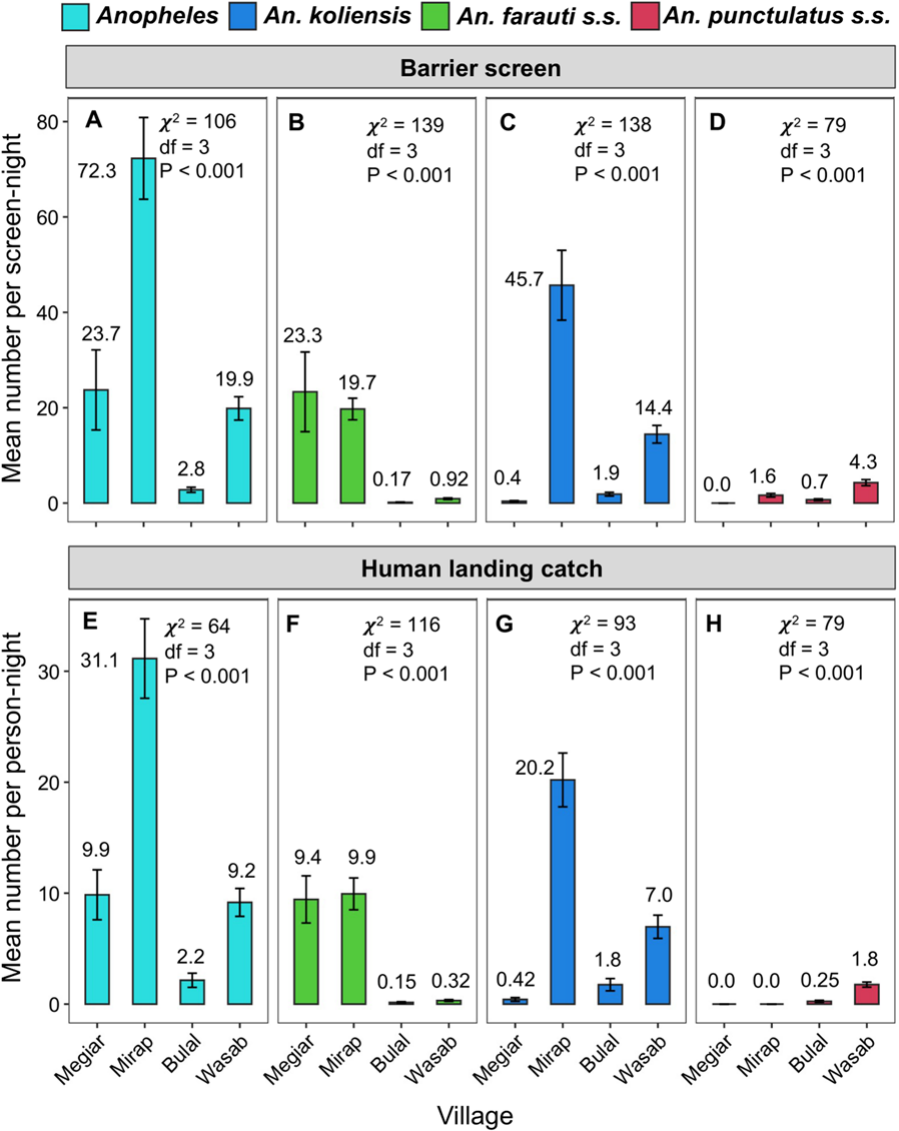
Bar plots showing the mean number (± standard error) of mosquitoes that were captured resting per barrier screen-night (**A**–**D**) and those that were captured biting per person-night (**E**–**H**) for *Anopheles* in general (skyblue), *An. farauti s.s.* (green)*, An. koliensis* (blue) and *An. punctulatus s.s*. (red) in each village. Numbers above the bars are the estimated means. The results of Kruskal–Wallis tests of variation in mosquito resting or biting rates among villages are shown inside the plot

The barrier screen resting rates for *Anopheles* in general varied significantly among villages (Kruskal–Wallis test: P < 0.001; Fig. 3A). It was highest in Mirap (mean = 72.3 mosquitoes per screen-night) followed by Megiar (mean = 23.7 per screen night), Wasab (mean = 19.9 per screen-night), and Bulal (mean = 2.8 per screen night) in decreasing order (Fig. 3A). Quantification of resting rates was also performed at the vector species level, but for *An. farauti s.s., An. koliensis* and *An. punctulatus s.s.* only; the other two species were ignored because of low numbers. The resting rates of all three species varied significantly among the villages (Kruskal–Wallis tests: P < 0.001; Fig. 3B–D). The resting rate of *An. farauti s.s.* (Fig. 3B) was higher in the coastal villages Megiar (mean = 23.3 per screen-night) and Mirap (mean = 19.7 per screen-night) than the inland villages Bulal (mean = 0.17 per screen-night) and Wasab (mean = 0.92 per screen night). The resting rate of *An. koliensis* (Fig. 3C) was highest in Mirap (mean = 45.7 per screen-night) followed by Wasab (mean = 14.4 per screen-night) which was *ca.* three-fold lower than Mirap. The other two villages had very low *An. koliensis* resting rates (mean ≤ 1.9 per screen-night). The resting rates of *An. punctulatus s.s.* (Fig. 3D) was highest in Wasab (mean = 4.3 per screen-night), followed by Mirap (mean = 1.6 per screen-night), and then by Bulal (mean = 0.7 per screen-night). This species was not observed in the BSS collections in Megiar.

The biting rates were also calculated for *Anopheles* in general and the three main vector species (Fig. 3E–H) in the villages. The biting rates of *Anopheles* as well as the three vector species varied significantly among the villages (Kruskal–Wallis tests: P < 0.001; Fig. 3E–H). The *Anopheles* biting rate (Fig. 3E) was highest in Mirap (31.1 per person-night) followed by Megiar (9.9) and Wasab (9.2) which had similar rates and were both *ca.* three-fold lower than Mirap, followed by Bulal (2.2) which was 14-fold lower than Mirap (Fig. 3E). Of the two main vector species in Mirap, *ca.* two-thirds of the *Anopheles* bites were delivered by *An. koliensis*, and one-third of the bites was delivered by *An. farauti s.s*. Almost all the *Anopheles* bites in Megiar were delivered by *An. farauti s.s.* and almost all the bites in Bulal were delivered by *An. koliensis*. Of the two main vectors in Wasab, *ca*. four-fifths of the *Anopheles* bites were delivered by *An. koliensis*, and *ca*. one-fifth of the bites were delivered by *An. punctulatus s.s.*

### Within-village spatial distribution of vectors

The results for tests of spatial variation in the frequency of mosquitoes sampled in houses within villages are shown for *An. koliensis* in three villages, *An. farauti s.s.* in two villages and *An. punctulatus s.s.* in one village (Table 1). These six vector populations had sufficient mosquito numbers for the analysis; the other populations were ignored because of low numbers. The index of dispersion was significantly greater than 1.0 for all six populations in both indoor and outdoor collections (Chi-square tests, P < 0.001; Table 1), which indicates a clustered rather than random or uniform spatial distribution of vectors.

**Table 1:** Mean, variance and index of dispersion of indoor and outdoor mosquito numbers in houses, along with the results of *χ*^2^ test of departure of index of dispersion from 1.0 for six vector populations

Village	Vector	Location	*n*	Mean	Var	ID	*χ*^2^	df	P
Bulal	*An. koliensis*	Indoor	10	5.0	62.7	12.5	112.8	9	< 0.001
		Outdoor	10	2.0	12.7	6.3	57	9	< 0.001
Megiar	*An. farauti s.s*	Indoor	12	9.4	278.4	29.6	325.3	11	< 0.001
		Outdoor	12	28.3	1199.3	42.3	465.6	11	< 0.001
Mirap	*An. farauti s.s*	Indoor	12	18.3	337.5	18.5	203.4	11	< 0.001
		Outdoor	12	21.5	108.6	5.1	55.6	11	< 0.001
	*An. koliensis*	Indoor	12	32.1	686.6	21.4	235.4	11	< 0.001
		Outdoor	12	48.8	766.2	15.7	172.9	11	< 0.001
Wasab	*An. koliensis*	Indoor	12	18.25	195.1	10.7	117.6	11	< 0.001
		Outdoor	12	37.5	1213.2	32.4	355.9	11	< 0.001
	*An. punctulatus s.s*	Indoor	12	5.6	19.4	3.5	38.1	11	< 0.001
		Outdoor	12	8.5	44.1	5.2	57.1	11	< 0.001

*Key: n*, number of houses; Var, variance; ID, index of dispersion; df, degrees of freedom = *n*–1; P, p-values associated with the *χ*^2^ tests

### Nocturnal, temporal biting patterns of vectors

**Fig. 4:**
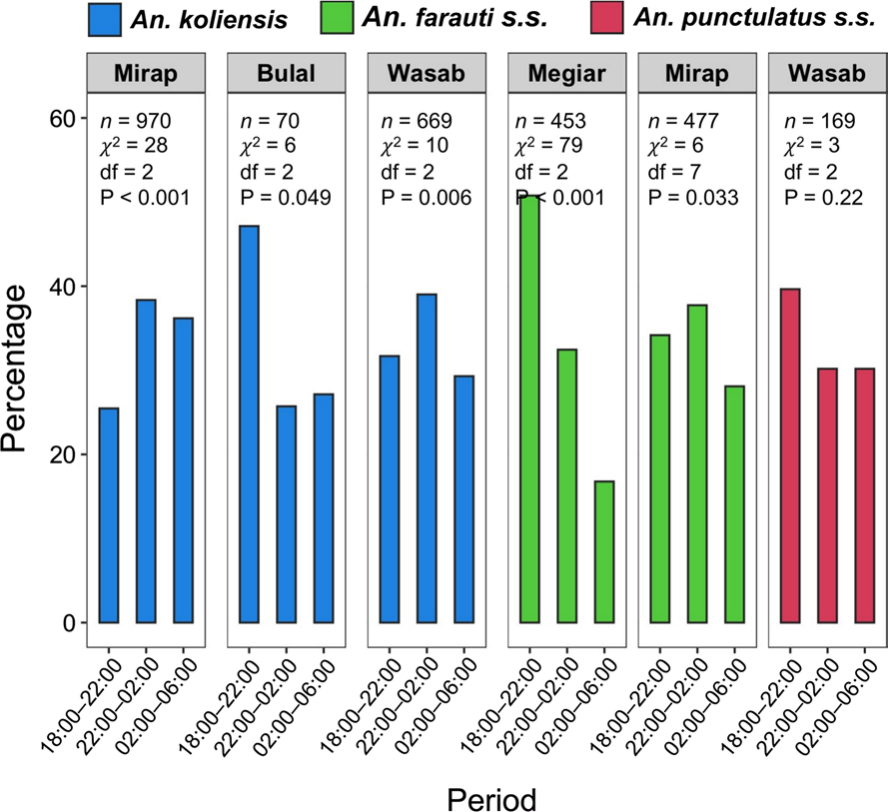
Bar plots showing the proportion of biting mosquitoes in three periods of the night for six vector populations: *An. koliensis* (blue) in three villages, *An. farauti s.s.* (green) in two villages and *An. punctulatus s.s.* (red) in one village. Mosquito sample size (*n*) and result of Chi-square test of variation in mosquito proportion among the periods are shown for each population inside the plots

The percentage of mosquitoes collected in the three periods of the night were calculated for the six vector populations (Fig. 4). Significant variation among the three periods was observed in all the populations (Chi-square tests: P < 0.05; Fig. 4) except for *An. punctulatus s.s*. in Wasab (P = 0.22). For *An. koliensis* in Bulal, *An. farauti s.s.* in Megiar and *An. punctulatus s.s*. in Wasab, most of the vector bites occurred in the evening (18:00–22:00), whereas for the other three populations, most of the bites occurred in the second period (22:00–02:00). The proportion of total bites that occurred in the evening ranged from 25.5 to 50.8% among the six populations (Fig. 4).

### Indoor and outdoor biting patterns of vectors

**Fig. 5:**
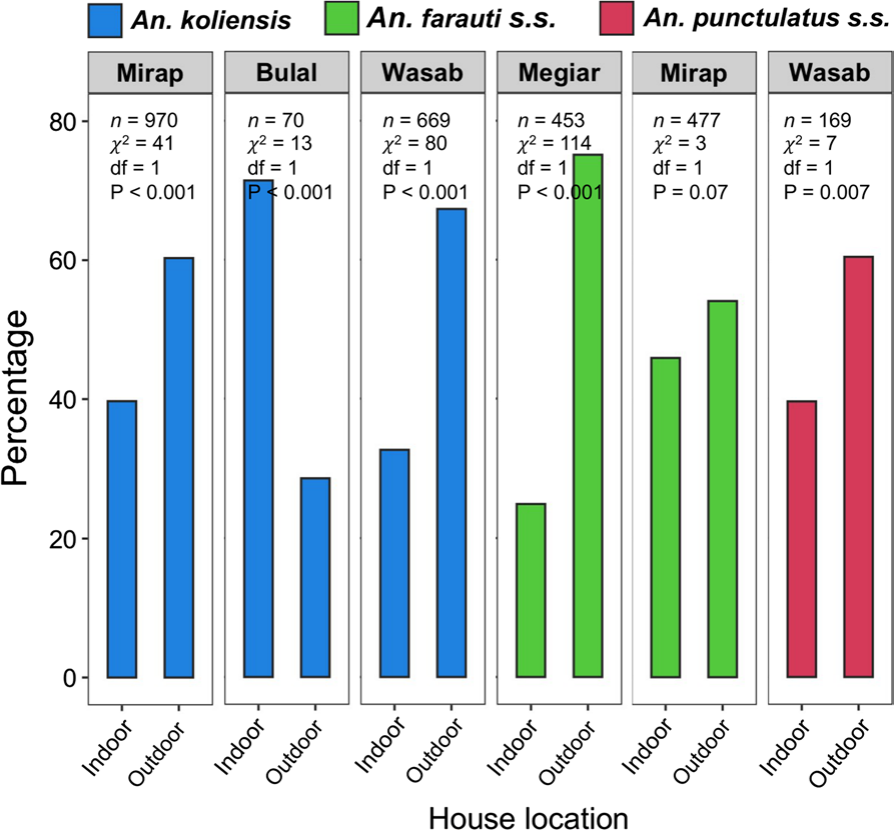
Bar plots showing the proportion of biting mosquitoes inside and outside houses for six vector populations: *Anopheles koliensis* (blue) in three villages, *Anopheles farauti s.s*. (green) in two villages and *Anopheles punctulatus s.s*. (red) in one village. Mosquito sample size (*n*) and results of Chi-square tests of variation in mosquito proportion between indoor and outdoor collections for each population are shown inside the plot

Proportionally, more mosquitoes (54.1–75.1%) were collected outdoors than indoors for all vector populations except *An. koliensis* in Bulal where the opposite outcome was observed (Fig. 5). Chi-square tests of proportions detected significant variation between indoor and outdoor mosquito numbers for all the populations (P < 0.05; Fig. 5) except for *An. farauti s.s.* in Mirap which was not significant.

### Sporozoite rates

The sporozoite rates for *Anopheles* are presented for each village in Table 2. Among villages, the sporozoite rates ranged from 0.0023 to 0.014 for *P. falciparum*, 0–0.0042 for *P. vivax* and 0.0034–0.0233 for *Plasmodium* in general (i.e., any species). *P. falciparum* was detected in *Anopheles* mosquitoes from all the villages. *P. vivax* was detected in *Anopheles* mosquitoes from all the villages except Bulal. Except in Megiar where the sporozoite rate in *Anopheles* was the same for both malaria species, the sporozoite rate of *P. falciparum* was two to seven-fold higher than that of *P. vivax* in the other three villages. The sporozoite rates of *P. falciparum*, *P. vivax* and *Plasmodium* in general for each vector species in each village were also calculated and are presented here (Table 3) but are not discussed further.

**Table 2:** Sporozoite rates of *P. falciparum*, *P. vivax* and *Plasmodium* in general in samples of *Anopheles* mosquitoes in general in each village

Village	*P. falciparum* *n* (S)	*P. vivax* *n* (S)	*Plasmodium* *n* (S)	Total tested mosquitoes
Megiar	2 (0.0042)	2 (0.0042)	4 (0.0084)	473
Mirap	21 (0.014)	3 (0.0020)	24 (0.016)	1495
Bulal	2 (0.0233)	0 (0)	2 (0.0233)	86
Wasab	2 (0.0023)	1 (0.0011)	3 (0.0034)	880

Values outside parentheses are number of sporozoite positive mosquitoes and inside parentheses are the sporozoite rates (S)

**Table 3:** Sporozoite rates of *P. falciparum*, *P. vivax* and *Plasmodium* in general in samples of *Anopheles* species in each village

Village	Vector	*P. falciparum* *n* (S)	*P. vivax* *n* (S)	*Plasmodium* *n* (S)	Total tested mosquitoes
Megiar	*An. farauti s.s*	2 (0.0044)	2 (0.0044)	4 (0.0088)	453
	*An. koliensis*	0 (0)	0 (0)	0 (0)	20
Mirap	*An. bancroftii*	0 (0)	0 (0)	0 (0)	2
	*An. farauti s.s*	4 (0.0084)	2 (0.0042)	6 (0.0126)	477
	*An. koliensis*	17 (0.0175)	1 (0.001)	18 (0.0186)	970
	*An. longirostris*	0 (0)	0 (0)	0 (0)	46
Bulal	*An. farauti s.s*	0 (0)	0 (0)	0 (0)	6
	*An. koliensis*	2 (0.0286)	0 (0)	2 (0.0286)	70
	*An. punctulatus s.s*	0 (0)	0 (0)	0 (0)	10
Wasab	*An. farauti s.s*	0 (0)	0 (0)	0 (0)	31
	*An. koliensis*	2 (0.003)	0 (0)	2 (0.003)	669
	*An. longirostris*	0 (0)	1 (0.0909)	1 (0.0909)	11
	*An. punctulatus s.s*	0 (0)	0 (0)	0 (0)	169

Values outside parentheses are number of sporozoite positive mosquitoes and inside parentheses are the sporozoite rates (S)

### Entomological inoculation rates

**Fig. 6:**
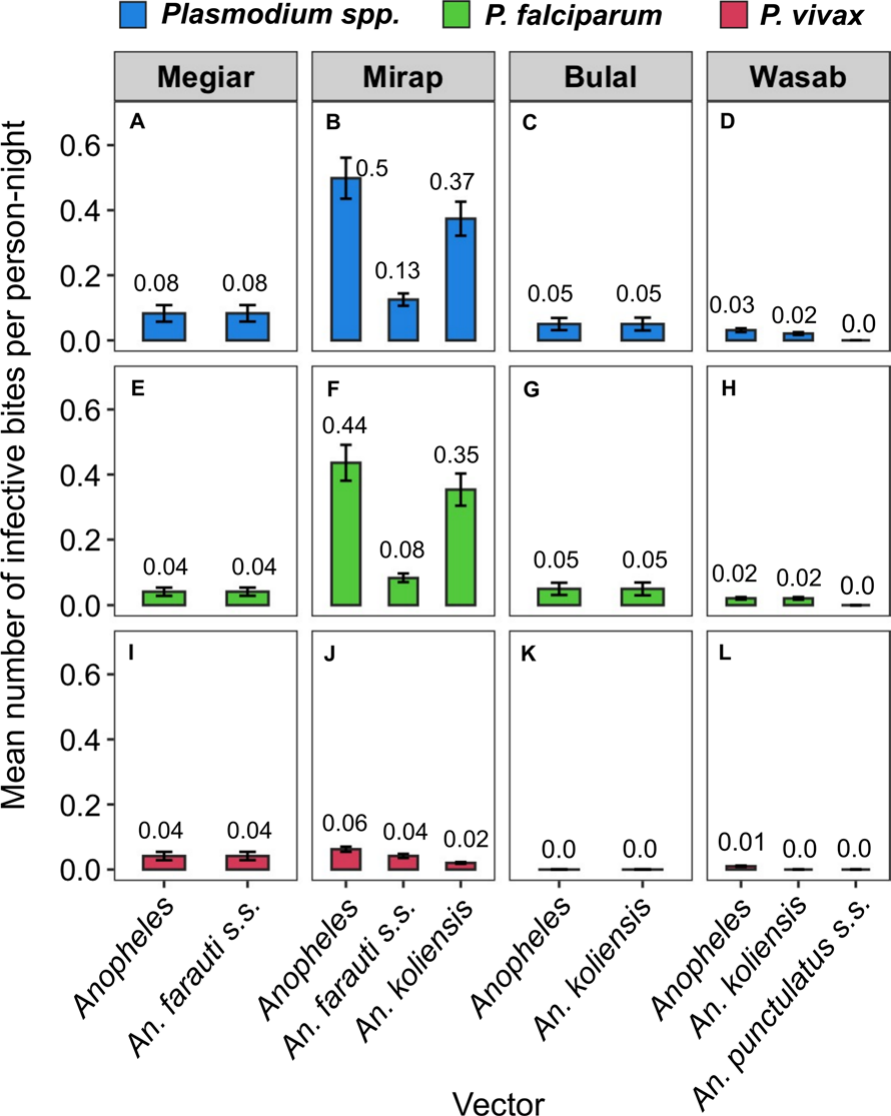
Bar plots showing the mean number (± standard error) of infective vector bites per person-night for *Plasmodium* in general (**A**–**D**, blue), *P. falciparum* (**E**–**H**, green) and *P. vivax* (**I**–**L**, red). These quantities are shown for *Anopheles* in general (first bar in each panel) and the predominant vector species (second to third bars in each panel) in each village. Numbers above the bars are the estimated means

The EIR varied among the villages for *Plasmodium* in general (Fig. 6A–D) as well as for *P. falciparum* (Fig. 6E–H) and *P. vivax* (Fig. 6 I–L). The EIR of *Plasmodium* in general by *Anopheles* in general was the highest in Mirap (0.5 infective bites per person-night). This was followed by Megiar (0.08 per person-night), which was six-fold lower than Mirap, followed by Bulal (0.05 per person-night) which was tenfold lower than Mirap, and then Wasab (0.03 per person-night) which was 17-fold lower than Mirap. The EIR of *P. falciparum* by *Anopheles* was highest in Mirap (0.44 infective bites per person-night), followed by Bulal (0.05 per person-night) and Megiar (0.04 per person-night), which were nine-fold and 11-fold, respectively, lower than Mirap, and then by Wasab (0.02 per person-night) which was *ca.* 22-fold lower than Mirap. For *P. vivax*, the EIR by *Anopheles* was highest in Mirap (0.06 infective bites per person-night), followed by Megiar (0.04 per person-night), which was 1.5-fold lower than Mirap, then by Wasab (0.01 per person-night) which was six-fold lower than Mirap, and then by Bulal (zero per person-night). The EIR of the dominant vector species in each village were also calculated and are presented in Fig. 6 but are not discussed further.

## Discussion

An important observation of the current study was that despite nearly a decade of an intensive LLIN-based vector control programme and high rates of bed net usage in the study villages, malaria continued to be transmitted at relatively high intensities in these villages. A study by Reimer et al. [11] in three of the four villages investigated here (Megiar, Mirap, Wasab) observed a 12- to 15-fold reduction in malaria transmission intensity one year after the roll-out of the LLIN programme in 2009. There is no published study that evaluated malaria transmission in these or other villages in the coastal areas of Madang in the intervening period (5 years) between Reimer et al. [11] and the current study. The nightly EIR for *Plasmodium* in general in the three villages in the current study (0.03–0.5 infective *Anopheles* bites per person-night) were 0 to 16-fold higher than in 2010 (0.03–0.04 infective *Anopheles* bites per person-night), a year after the LLIN roll-out [11]. It is worth noting that malaria transmission intensity reported in Reimer et al. [11] was expressed in terms of annual EIR but were converted to nightly EIR here for comparison with the current data. The observations between the previous and current studies indicate an increase in malaria transmission intensity in recent years after a period of decline following the roll-out of the LLIN campaign. However, this assertion is considered here only as a plausible observation that needs to be confirmed. Unlike Reimer et al. [11] whose estimates of transmission intensity were based on longitudinal mosquito sampling throughout the year to account for temporal variation associated with patterns of rainfall, the estimates in the current study may be biased as they were based on mosquito data from a single time point. Nevertheless, the assertion regarding increased transmission intensity is consistent with epidemiological trends which also show an increase in infection prevalence in humans in recent years in these study villages [37] as well as nationally [36]. Also, given nearly a decade of continued, intensive vector control in PNG, including Madang, malaria transmission was expected to be maintained at intensities much lower than the 2010 estimates and fluctuates only slightly even during periods of high rainfall. The result of the current study was inconsistent with this expectation and tended to support a plausible increase in transmission intensity.

Vector abundance was also high relative to previous estimates. In Megiar, Mirap and Wasab, the human biting rates of *Anopheles* in general ranged from 9.2 to 31.1 bites per person-night and the barrier screen resting rates ranged from 19.9 to 72.3 mosquitoes per screen-night. The biting rates of the three villages reported here were two- to three-fold higher than in 2010 (3–16 bites per person-night) [11]. It is worth noting that the 2010 biting rates represented 2.0–3.7-fold reduction compared to pre-LLIN estimates in 2008–2009 [11]. The resting rates in Mirap (72.3 mosquito per screen-night) and Wasab (19.9 mosquito per screen-night) were both two-fold higher than the resting rates in these villages in 2012 [51]. The comparison between the current and previous studies tended to show an increase in vector abundance in recent years. As discussed in the introduction, insecticide resistance is an unlikely factor to cause the observed increase in mosquito abundance. Also, to the best of our knowledge and observation, there was no major change in land use such as large-scale agricultural activities, mining or logging in or around the study area after the LLIN campaign was implemented that would result in increased production of mosquitoes compared to pre-LLIN period. However, as with transmission intensity, the estimates of vector abundance in the current study were based on mosquito data from a single time point and may be biased by temporal, seasonal factors. For example, except for six of the 12 mosquito-sampling nights in Wasab which occurred in the dry season, sampling in all villages including the other six nights in Wasab occurred during the wet season where mosquito abundance is usually high. Therefore, the assertion regarding increased vector abundance can only be considered here as a plausible observation that requires a more robust, longitudinal study to confirm. Nevertheless, this assertion is consistent with the prediction that the distribution of poor-quality LLINs in PNG, including Madang [27], results in increased vector abundance and, consequently, malaria transmission intensity.

High vector abundance and transmission intensity (relative to the results observed immediately after the LLIN programme [11]) in the presence of an intensive LLIN-based vector control programme and high bed net usage rates can be caused by various factors. In addition to the use of poor-quality LLINs, outdoor and early biting behaviours of vectors may also be contributing factors. Unlike some African vector populations, which remain inside human dwellings after a blood meal, PNG vectors are typically exophilic [64, 65]. This means that PNG vectors rest as adults in the vegetation, enter a village to find vertebrate hosts, and exit the village after taking a blood meal to rest in the vegetation [64]. By feeding on humans outdoors or in the evening, the vectors can return to the vegetation without coming in contact with the bed nets and by the same logic, indoor residual sprays which are deployed inside houses [47, 48, 66]. In the current study, more (> 54%) of the vectors encountered humans outdoors than indoors in all villages except Bulal which had the opposite outcome. Similarly, a considerable proportion (25.5–50.8%) of the vectors in all the villages encountered human hosts in the evening. These results show that a high proportion of vectors evaded LLINs by encountering humans outdoors and in the evening and may be contributing to high vector abundance and malaria transmission in these villages. This assertion is supported by the finding of a separate study which showed that shift in the peak biting time of vectors from late night to early hours of the evening resulted in increased biting rates (a measure of vector abundance) and risk of exposure to infective bites to pre-LLIN levels in two inland villages in Madang despite high LLIN usage rates in these villages during the study [12].

Another notable observation was high degree of variability in the entomological quantities investigated in the current study. Presence of multiple vector species that vary in their relative composition within and among villages, even neighbouring ones, is a commonly observed phenomenon in PNG [6–11, 15, 34, 51, 67–71]. This variation is caused by the distribution and abundance of their preferred larval habitat types [72]. For example, the ability of *An. farauti s.s.* to tolerate brackish water allows this vector to occupy this type of habitats without competition from the other vector species, making it the dominant vector in coastal plain and islands areas where such habitats are abundant [15, 72, 73]. Its ability to also utilize freshwater allows it to be found sporadically, and in low abundance, in inland areas [14]. Adaptation of *An. punctulatus s.s.* and *An. koliensis* to freshwater allows them to occupy this habitat type with very little competition from *An. farauti s.s.*, making them the dominant vectors in inland areas where freshwater habitats are more abundant [15, 72]. Their presence in coastal areas is associated with availability of freshwater bodies both transient, e.g., rain and riverine puddles, and semi-permanent, e.g., shallow ground pools and swamps [15, 72]. *An. koliensis*, a strongly anthropophilic species, was the most abundant vector in all the villages (63.7–73.8%), except Megiar where *An. farauti s.s.* was more abundant. The high abundance of *An. koliensis* observed here might indicate reduced effectiveness of the LLINs considering that this vector was greatly affected by the roll-out of the LLINs making it less or the least abundant compared to the other vector species in Madang and other parts of PNG [11, 34]. However, the abundance of *An. koliensis* observed here might be caused by temporary seasonal condition favourable to the production of this species (e.g., during wet season) at the time this study was conducted and not a long-term phenomenon associated with LLIN effectiveness.

Vector abundance and malaria transmission intensity also varied significantly among the villages. The barrier screen resting rates of *Anopheles* in general among villages ranged from 2.8 mosquitoes per screen-night in Bulal to 72.3 per screen-night in Mirap, a 26-fold difference in mosquito abundance. Their biting rates ranged from 2.2 bites per person-night in Bulal to 31.1 bites per person-night in Mirap, a 14-fold difference in mosquito abundance. Variation in vector abundance among villages in close spatial proximity observed here was consistent with similar observations in Madang villages in previous studies [11, 51], suggesting that heterogeneity in vector abundance is a common phenomenon in PNG. As human biting rates directly affect the estimates of transmission intensity, it was not surprising that the EIR of *Plasmodium* in general varied from 0.03 infective *Anopheles* bites per person-night in Wasab to 0.5 infective *Anopheles* bites per person-night in Mirap, a 17-fold difference in transmission intensity. For *P. falciparum*, this quantity ranged from 0.02 infective *Anopheles* bites per person-night in Wasab to 0.44 infective *Anopheles* bites per person-night in Mirap, a 22-fold difference in transmission intensity. For *P. vivax*, this quantity ranged from zero infective *Anopheles* bites per person-night in Bulal to 0.06 infective *Anopheles* bites per person-night in Mirap. These results were consistent with those of other studies, which also found great disparity in transmission intensities among villages in the coastal and highlands provinces of PNG [9, 11, 34].

Entomological heterogeneity among villages, like the ones observed in the current study, is important as it can complicate vector control programmes, and allow malaria transmission to persist [74]. When different areas within a province or country are homogeneous in entomological factors such as vector species composition, their abundance, biting patterns, host selection and other ecological attributes, malaria control based on methods that target the vectors such as the LLINs are generally easier to achieve. This is because a control method that is effective against vectors in one area can also be effective on those in other areas, resulting in uniform impact on malaria epidemiology throughout the country. In contrast, malaria control can be difficult to achieve when vectors in different areas vary in attributes. This is because a control method that is effective against vectors in one area may not be effective against vectors in other areas, resulting in heterogeneous impact on malaria epidemiology throughout the country. This causes malaria to persist in some areas of the country and potential for resurgence in areas where it was successfully controlled. Similarly, in an area where multiple vector species coexist but vary in attributes, a control method may work on some species but not others, causing malaria to persist in that area.

Vector abundance not only varied among but also within the study villages. In all four villages, the frequency of mosquitoes in different houses within a village did not fit a random Poisson distribution. Instead, the data fit a clustered distribution. This means that some locations within a village had higher mosquito abundance than most other locations. While it is possible that the clustered spatial distribution of mosquito abundance can be caused in part by collector bias because collectors were not rotated among houses, it could also be caused by various other factors. One potential factor is distance from mosquito oviposition and resting habitats. That is, parts of a village that are closer to these habitats are more likely to have higher adult mosquito abundance than those further away. In this study, data on larval habitats and resting sites were not obtained to test this prediction. However, other studies have shown that houses within villages that are closer to larval habitats tend to have high adult mosquito abundance than those further away [75, 76]. Note that locations with high abundance of vectors equates to high human biting rates and transmission intensities in those areas and might explain the within-village spatial variation in the risk of malaria infection observed in Megiar and Mirap [37]. Also, epidemiological models have shown that the basic reproduction rate and vectorial capacity of malaria both increase when the biting rates vary spatially [49, 77–79]. Because transmission increases with both quantities, spatial heterogeneity in vector abundance in these villages might help cause malaria to persist even when the LLIN program is in place [79].

This study has one caveat that needed to be addressed. Unlike the immunologic assays that test for sporozoite-specific stage of malaria parasites in mosquitoes [80–83], the PCR method used here is not sporozoite-specific; it can detect any stage of the parasites. Thus, it is possible that some of the malaria-positive mosquitoes might have carried non-sporozoite stages of the parasite. This could result in overestimation of the sporozoite rates. As sporozoite rate was used to estimate the EIR, it is possible that the estimates of this quantity could also be overestimated. This problem was minimized by restricting the PCR tests to the heads and thoraces of the mosquitoes where only the sporozoites inhabit and not the abdomen where the non-sporozoite stages inhabit [84].

## Conclusions

The results of this study indicate that vector abundance and malaria transmission in the coastal areas is Madang and likely in other parts of PNG as well may have increased in recent years after a period of decline after the roll-out of the LLIN campaign in 2009. However, because this study was conducted in a single time point, the level of certainty about the increasing trend in vector abundance and transmission intensity is weak and requires a more robust, longitudinal approach to confirm. Nevertheless, the estimates of both quantities were higher than the results observed immediately after the LLIN programme [11]. This indicates that although the LLINs provide some level of protection against malaria, their effectiveness was limited by other factors such as outdoor and early biting behaviors of the vectors and the use of poor-quality LLINs. This study also observed a high degree of heterogeneity in vector abundance and species composition among and within villages. As entomological heterogeneity complicates vector control programmes such as the LLINs, this factor must be taken into consideration when planning such programmes in PNG.

## Supplementary Information

**Additional file 1. Table S1. **Composition of Anopheles species in mosquito samples from four different villages. Values outside parentheses are mosquito numbers (n) and inside parentheses are percentages of column totals.**Table S2.** Composition of Anopheles species in mosquito samples collected in coastal and inland environments. Values outside parentheses are mosquito numbers (n) and inside parentheses are percentages of column totals.

## Data Availability

Data supporting the conclusions of this article are included within the article and its additional files.

## Abbreviations

ACT: Artemisinin-based combination therapy
BSS: Barrier screen sampling
DNA: Deoxyribonucleic acid
HLC: Human landing catch
LLINs: Long-lasting insecticidal bed nets
PCR: Polymerase chain reaction
PNG: Papua New Guinea
rRNA: Ribosomal ribonucleic acid
