# Supplementary material for: Vector composition, abundance, biting patterns and malaria transmission intensity in Madang, Papua New Guinea: assessment after 7 years of an LLIN-based malaria control programme
Source: Malar J. 2022 Jan 5;21:7. doi: 10.1186/s12936-021-04030-4 (PMC8729043; doi:10.1186/s12936-021-04030-4)
Supplement: Supplementary file 1 — Additional file 1. Table S1. Composition of Anopheles species in mosquito samples from four different villages. Values outside parentheses are mosquito numbers (n) and inside parentheses are percentages of column totals.Table S2. Composition of Anopheles species in mosquito samples collected in coastal and inland environments. Values outside parentheses are mosquito numbers (n) and inside parentheses are percentages of column totals. [file 12936_2021_4030_MOESM1_ESM.docx]

**Table S1.** Composition of *Anopheles* species in mosquito samples from four different villages. Values outside parentheses are mosquito numbers (*n*) and inside parentheses are percentages of column totals.

| Vector | Megiar  *n* (%) | Mirap  *n* (%) | Bulal  *n* (%) | Wasab  *n* (%) |
| --- | --- | --- | --- | --- |
| *An. bancroftii* | 0 (0.00) | 142 (2.86) | 0 (0.00) | 0 (0.00) |
| *An. farauti s.s.* | 1573 (97.58) | 1424 (28.68) | 14 (6.36) | 119 (4.27) |
| *An. koliensis* | 39 (2.42) | 3163 (63.71) | 160 (72.73) | 2055 (73.76) |
| *An. longirostris* | 0 (0.00) | 157 (3.16) | 2 (0.91) | 31 (1.11) |
| *An. punctulatus s.s.* | 0 (0.00) | 79 (1.59) | 44 (20.00) | 581 (20.85) |

**Table S2.** Composition of *Anopheles* species in mosquito samples collected in coastal and inland environments. Values outside parentheses are mosquito numbers (*n*) and inside parentheses are percentages of column totals.

| Vector | Coastal  *n* (%) | Inland  *n* (%) |
| --- | --- | --- |
| *An. bancroftii* | 142 (2.16) | 0 (0.00) |
| *An. farauti s.s.* | 2997 (45.57) | 133 (4.42) |
| *An. koliensis* | 3202 (48.68) | 2215 (73.69) |
| *An. longirostris* | 157 (2.39) | 33 (1.10) |
| *An. punctulatus s.s.* | 79 (1.20) | 625 (20.79) |
